# The first case of paraneoplastic pemphigus positive for IgG autoantibodies against integrin α6^☆^

**DOI:** 10.1016/j.abd.2024.04.016

**Published:** 2025-01-22

**Authors:** Ying Li, Yunmei Dong, Xin Zeng, Wei Li, Yu Zhou, Xiaoguang Li

**Affiliations:** a State Key Laboratory of Oral Diseases, National Clinical Research Center for Oral Diseases, Chinese Academy of Medical Sciences Research Unit of Oral Carcinogenesis and Management, West China Hospital of Stomatology, Sichuan University, Chengdu, China; b Department of Dermatovenereology, Rare Disease Center, West China Hospital, Sichuan University, Chengdu, China; c School of Public Health and Laboratory Medicine, Hunan University of Medicine, Huaihua, China; d Department of Laboratory Medicine, Chronic Disease Research Center, Medical College, Dalian University, Dalian, China

*Dear Editor,*

Paraneoplastic Pemphigus (PNP) is a rare Autoimmune Bullous Disease (AIBD) associated with neoplasm.^1^ The following features can be used as references for a potential diagnosis of PNP, including (i) Mucous lesions, (ii) Histologic characteristics indicative of acantholysis or lichen planus, (iii) Positive for autoantibodies against plakin proteins, and (iv) Neoplasm.^2^ In PNP, autoantibodies against diverse Basement Membrane Zone (BMZ) autoantigens, such as BP180, Laminin (LM)-332, and LMγ1, have been identified.^2^, ^3^ Given the high mortality rate of PNP, particularly in cases featuring bronchiolitis obliterans, prompt and accurate diagnosis assumes paramount importance.^2^

A 34-year-old female visited our institution on Day 0 with a one-month history of oral white patches. Histopathological analysis of a tongue biopsy revealed mucosal epithelial hyperplasia and atrophy, alongside hydropic degeneration of the basal cells and inflammatory infiltration of lymphocytes and plasma cells (Fig. 1A), indicating lichen planus. On day 118, the patient exhibited a recurrence of oral ulceration, erosion, blisters, and white stripes, accompanied by tenderness and a positive Nikolsky sign (Fig. 1B), indicative of potential AIBD. Histopathological examination of a cheek biopsy showed the features of lichen planus (data not shown). Direct immunofluorescence revealed positive intercellular staining of IgG (Fig. 1C) and C3, but not IgA and IgM (Table S1).

**Figure 1:**
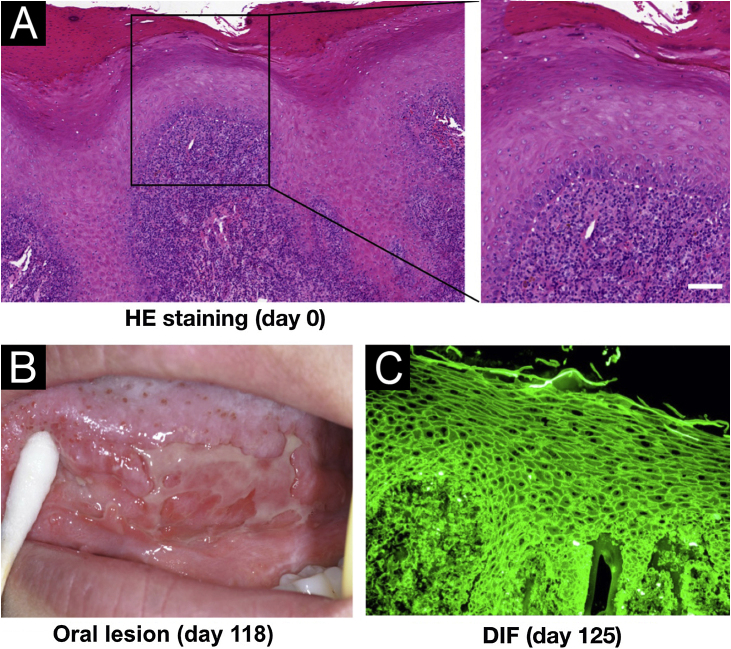
Clinical and pathological features of this patient. (A) Histopathological features for the biopsy of the right margin of tongue with white patch (Hematoxylin & eosin staining, scar bar = 50 μm). (B) Oral lesions. (C) Direct Immunofluorescence (DIF) showed positive intercellular staining of IgG.

Indirect Immunofluorescence (IIF) using normal human skin showed negative IgG staining (Fig. 2A) but positive intercellular staining of IgA (Fig. 2B). Rat bladder IIF showed IgG staining on granular cell surface and BMZ (Fig. 2C). IIF using 1M NaCl-split normal human skin (ssIIF) showed IgG and IgA staining in both epidermal and dermal sides (Fig. 3A and 3 B). Immunoblotting (IB) of epidermal extract detected IgG and IgA autoantibodies against envoplakin and periplakin (Fig. 4A). IB of dermal extract detected IgG and IgA anti-LMγ1 autoantibody (Fig. 4B). IB of integrin α6β4 Recombinant Protein (RP) detected IgG anti-integrin α6 autoantibody (Fig. 4C). IB of LM332 RP and in house LM332 RP ELISA detected no autoantibodies against LM332 (data not shown).^4^ Furthermore, ELISAs confirmed the presence of IgG autoantibodies against BP230, but not those against Desmoglein (Dsg) 1, Dsg3, BP180, or type VII collagen (Fig. 4D). On day 125, the emergency of ocular erosion was observed for the first time (data not shown). A comprehensive summary of the serological and immunofluorescence findings of this case is presented in Table S1.

**Figure 2:**
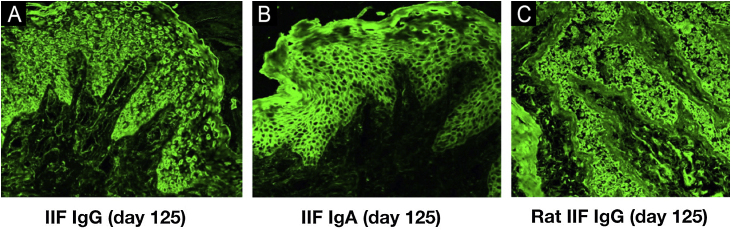
Indirect Immunofluorescence (IIF) results of this patient serum. IIF using normal human skin showed negative IgG staining (A) and positive intercellular staining of IgA (B). (C) IIF using rat bladder tissue showed IgG staining on granular cell surface and BMZ.

**Figure 3:**
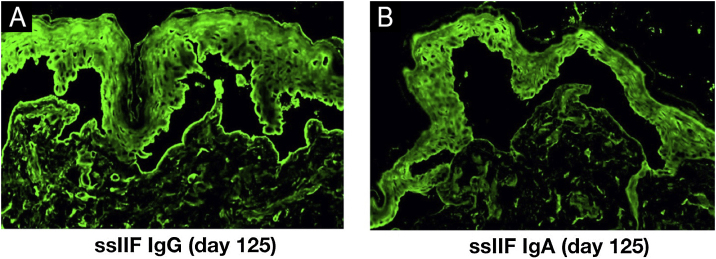
IIF using 1 M NaCl-split normal human skin (ssIIF) results of this patient serum. ssIIF showed both IgG (A) and IgA (B) staining in both epidermal and dermal sides.

**Figure 4:**
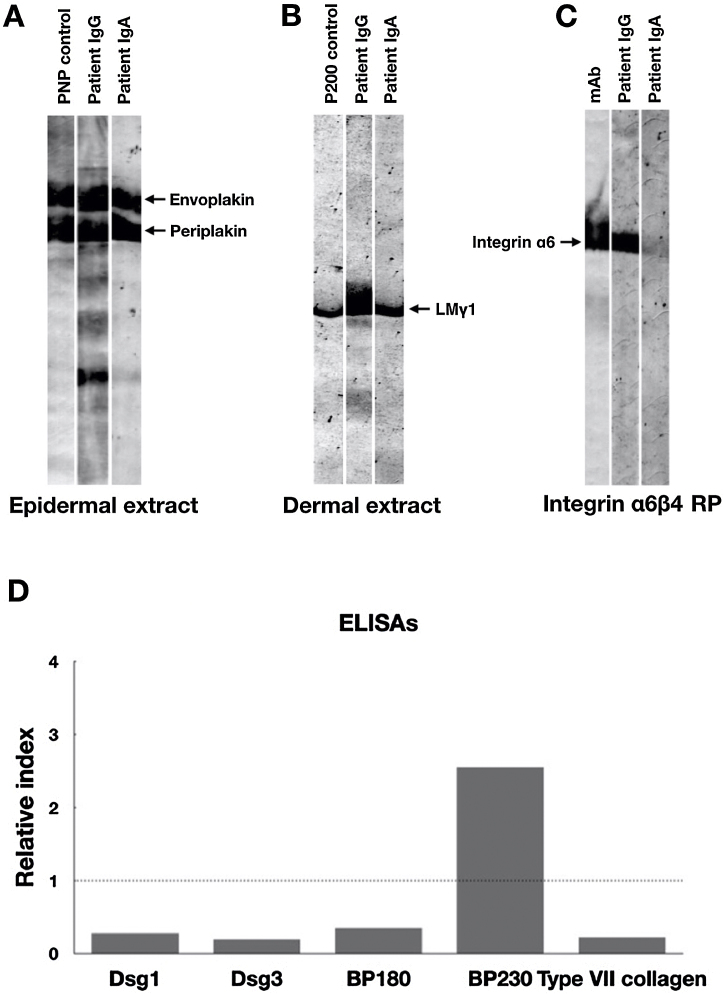
Immunoblotting and ELISA results of this patient serum. (A) Immunoblotting of epidermal extract showed positive IgG and IgA autoantibodies against both envoplakin and periplakin. (B) Immunoblotting of dermal extract showed positive IgG and IgA autoantibodies against Laminin (LM) γ1. (C) Immunoblotting of integrin α6β4 Recombinant Protein (RP) showed positive IgG autoantibodies against integrin α6. The integrin α6β4 RP was purchased from R&D systems (Minneapolis, MN, USA). mAb, a monoclonal antibody to integrin α6 (Beyotime, Shanghai, China). (D) Detection of IgG autoantibodies against Desmoglein (Dsg) 1, Dsg3, BP180, BP230 and type VII collagen by ELISAs using a commercially available kit (MBL, Japan). Only anti-BP230 autoantibodies were found to be positive. Dot lines indicate the cutoff value.

Based on the aforementioned data, this patient’s condition was suspected to be PNP. Subsequently, a Castleman tumor was found and surgically excised, with histopathological examination revealing follicular dendritic cell sarcoma. Additionally, bronchiolitis obliterans was confirmed via lung histopathology. After surgery, the patient’s symptoms were gradually relieved with appropriate therapy. A comprehensive overview of the clinical features and treatment regimen spanning from day 0 to day 332 are summarized in Table S2.

In the present case, early AIBD serological analyses suggested the possible diagnosis of PNP, which accelerated the discovery of the Castleman tumor, confirmed the PNP diagnosis, and resulted in subsequent adjustment of therapeutic strategies. This underscores the pivotal significance of early serological diagnosis of PNP.

During the progression of the disease, this patient presented mucosal lesions without concurrent skin lesions. Except for plakin proteins, this patient’s serum was also positive for autoantibodies against integrin α6, LMγ1, and BP230. To the best of our knowledge, this is the first reported case of PNP featuring anti-integrin α6 autoantibodies. Both integrin β4 and integrin α6 are considered important autoantigens for pure ocular Mucous Membrane Pemphigoid (MMP), although integrin α6 (30%) is less common than integrin β4 (60%).^5^ Moreover, both IgG and IgA autoantibodies against integrin α6 and/or integrin β4 have been detected in pure ocular MMP.^5^ Notably, integrin α6 is considered a principal autoantigen of oral MMP.^6^ Recently, our group reported an MMP case, which presented only mucosal lesions and was positive for only anti-LMγ1autoantibodies, indicating a potential role for anti-LMγ1 autoantibodies on mucous lesion development.^7^

The detection of anti-integrin α6 autoantibodies in our PNP case underscores the importance of incorporating both integrin α6 and integrin β4 in the diagnostic assessment of patients with this disease. Furthermore, we believe that the scientific investigation of the pathogenic role of autoantibodies targeting this molecule deserves more attention.

## Financial support

This work was sponsored by the Education Fund Item of Liaoning Province (LJKMZ20221843).

## Authors’ contributions

Ying Li: Drafting the article, revising it critically for important intellectual content, approved the final version to be published, full access to all of the data in the study, and took responsibility for the integrity of the data and the accuracy of the data analysis, collected case information and laboratory data, and analyzed the data.

Yunmei Dong: Drafting the article, revising it critically for important intellectual content, approved the final version to be published, collecting case information and laboratory data, and analyzing the data.

Xin Zeng: Drafting the article, revising it critically for important intellectual content, approving the final version to be published, collecting case information and laboratory data, and analyzing the data.

Wei Li: Drafting the article, revising it critically for important intellectual content, approving the final version to be published, collecting case information and laboratory data, and analyzing the data.

Yu Zhou: Drafting the article, revising it critically for important intellectual content, approving the final version to be published, full access to all of the data in the study, and took responsibility for the integrity of the data and the accuracy of the data analysis, conceived and designed the project, collected case information and laboratory data, and analyzed the data.

Xiaoguang Li: Drafting the article, revising it critically for important intellectual content, approving the final version to be published, full access to all of the data in the study and took responsibility for the integrity of the data and the accuracy of the data analysis, conceived and designed the project, collected case information and laboratory data, and analyzed the data.

## Conflicts of interest

None declared.
