# Supplementary material for: The first case of paraneoplastic pemphigus positive for IgG autoantibodies against integrin α6
Source: An Bras Dermatol. 2025 Jan 22;100(2):380–3. doi: 10.1016/j.abd.2024.04.016 (PMC11963105; doi:10.1016/j.abd.2024.04.016)
Supplement: Supplementary file 1 [file mmc1.docx]

**ABD-D-24-00149_Supplementary Material**

**Table S1** Serological and immunofluorescence features of this PNP case.

| **Detection Methods** | **Results** |
| --- | --- |
| DIF for keratinocyte surface/BMZ (IgG) | (+)/(-) |
| DIF for keratinocyte surface/BMZ (IgA) | (-)/(-) |
| DIF for keratinocyte surface/BMZ (IgM) | (-)/(-) |
| DIF for keratinocyte surface/BMZ (C3) | (+)/(-) |
| IIF for keratinocyte surface/BMZ (IgG) | (-)/(-) |
| IIF for keratinocyte surface/BMZ (IgA) | (+)/(-) |
| Rat IIF granular cell surface/BMZ (IgG) | (+)/(+) |
| ssIIF reactive with epidermal/dermal side (IgG) | (+)/(+) |
| ssIIF reactive with epidermal/dermal side (IgA) | (+)/(+) |
| IB of normal human epidermal extracts for envoplakin/ periplakin (IgG) | (+)/(+) |
| IB of normal human epidermal extracts for envoplakin/ periplakin (IgA) | (+)/(+) |
| IB of normal human dermal extract for 200 kDa LMg1 (IgG) | (+) |
| IB of normal human dermal extract for 200 kDa LMg1 (IgA) | (+) |
| IB of integrin α6β4 RP for integrin α6/β4 (IgG) | (+)/(-) |
| IB of integrin α6β4 RP for integrin α6/β4 (IgA) | (-)/(-) |
| IB of LM332 RP for LM332 (IgG) | (-) |
| ELISA of LM332 RP for LM332 (in house, IgG) | (-) |
| ELISA for desmoglein 1 (IgG) | (-) |
| ELISA for desmoglein 3 (IgG) | (-) |
| ELISA for BP180 (IgG) | (-) |
| ELISA for BP230 (IgG) | (+) |
| ELISA for type VII collagen (IgG) | (-) |

**Table S2** Summary of clinical, pathological and treatment data of this patient.

| **Days** | **Day 0** | **Day 118** | **Day 125** | **Day 167** | **Day 281** | **Day 318** | **Day 332** |
| --- | --- | --- | --- | --- | --- | --- | --- |
| **Diagnosis** | LP? | | LP,  PNP? | LP,  PNP,  Castleman? | LP,  PNP,  BO, FDCS | LP,  PNP,  BO, FDCS, bacterial pneumonia, hypokalemia | |
| **Clinical  features** | painless white patches | reoccurrence of white stripes, blister,  tongue ulceration, erosion on cheeks, Nikolsky sign (+), tenderness | ocular congestion,  and erosion, tongue ulceration, erosion on cheeks, Nikolsky sign (+), tenderness | ocular congestion, swollen face, hair loss, upper lip blister, tongue ulceration, erosion on cheeks, Castleman tumor | surgical removement of right subclavian mass， FDCS， BO | discharge from hospital | oral symptoms relieved gradually |
| **Treatment** | day 0: (1) Beta-Carotene Capsules, 6 mg per day, p.o.; (2) Dexamethasone sodium phosphate injection, 10-fold diluted, tid, external use.  day 85: (1) Prednisone Acetate Tablets, 30 mg per day, p.o.; (2) Dexamethasone sodium phosphate injection, 10-fold diluted, tid, external use; (3) 2% Sodium Bicarbonate Injection, tid, external use.  day 97/day 118: (1) Compound Betamethasone Injection, 1.2ml, submucosal injection only once; (2) Thalidomide Tablets, 50 mg per day, p.o.; (3) Dexamethasone sodium phosphate injection, 10-fold diluted, tid, external use.. | | day 125: (1) Prednisone Acetate Tablets, 40 mg per day, p.o.; (2) Potassium Chloride Sustained-release Tablets, 1 g per day, p.o.; (3) Calcium Carbonate and Vitamin D3 Tablets, 600 mg per day, p.o. (4) 2% Sodium Bicarbonate Injection, 30ml per day, tid, external use.  day 146/day 153: (1) Prednisone Acetate Tablets, 40 mg per day, p.o.; (2) Potassium Chloride Sustained-release Tablets, 1 g per day, p.o.; (3) Calcium Carbonate and Vitamin D3 Tablets, 600 mg per day, p.o.; (4) 2% Sodium Bicarbonate Injection, 30ml per day, tid, external use; (5) Dexamethasone sodium phosphate injection, 10-fold diluted, tid, external use; (6) Vitamin C Tablets, 0.6g per day, p.o..  day 160: (1) Prednisone Acetate Tablets, 35 mg per day, p.o.; (2) Potassium Chloride Sustained-release Tablets, 1 g per day, p.o.; (3) Calcium Carbonate and Vitamin D3 Tablets, 600 mg per day, p.o.; (4) 2%Sodium Bicarbonate Injection, 30ml per day, tid, external use; (5) Dexamethasone sodium phosphate injection, 10-fold diluted, tid, external use; (6) Vitamin C Tablets, 0.6g per day, p.o.. | day 167: (1) Prednisone Acetate Tablets, 40 mg per day, p.o.; (2) Potassium chloride sustained-release Tablets, 1 g per day, p.o.; (3) Calcium Carbonate and Vitamin D3 Tablets, 600 mg per day, p.o.;   day 206: drug discontinuance | day 281: drug discontinuance.  day 297: (1) Prednisone Acetate Tablets, 35 mg per day, p.o.; (2) Potassium Chloride Sustained-release Tablets, 1 g per day, p.o.; (3)Calcium Carbonate and Vitamin D3 Tablets, 600 mg per day, p.o.; (4) 2% Sodium Bicarbonate Injection, tid, external use;(5) Dexamethasone sodium phosphate injection, 10-fold diluted, tid, external use.  day 307: (1) Prednisone Acetate Tablets, 30 mg per day, p.o.; (2) Potassium Chloride Sustained-release Tablets, 1 g per day, p.o.; (3)Calcium Carbonate and Vitamin D3 Tablets, 600 mg per day, p.o.; (4) 2% Sodium Bicarbonate Injection, tid, external use; (5) Dexamethasone sodium phosphate injection, 10-fold diluted, tid, external use. | day 318: (1) Prednisone Acetate Tablets, 25 mg per day, p.o.; (2) Potassium Chloride Sustained-release Tablets, 1 g per day, p.o.; (3) Calcium Carbonate and Vitamin D3 Tablets, 600 mg per day, p.o.; (4) 2% Sodium Bicarbonate Injection, tid, external use; (5) Dexamethasone sodium phosphate injection, 10-fold diluted, tid, external use.  day 332: (1) Prednisone Acetate Tablets, 22.5 mg per day, p.o.; (2) Potassium Chloride Sustained-release Tablets, 1 g per day, p.o.; (3) Calcium Carbonate and Vitamin D3 Tablets, 600 mg per day, p.o.; (4) 2% Sodium Bicarbonate Injection, tid, external use; (5) Dexamethasone sodium phosphate injection, 10-fold diluted, tid, external use. | |

LP, lichen planus; PNP, paraneoplastic pemphigus; BO, bronchiolitis obliterans; FDCS, follicular dendritic cell sarcoma; p.o., oral administration; tid, three times/day.
